# Bioactives and Extracellular Enzymes Obtained from Fermented Macrofungi Cultivated in Cotton and *Jatropha* Seed Cakes

**DOI:** 10.3390/microorganisms10081670

**Published:** 2022-08-19

**Authors:** Joice Raísa Barbosa Cunha, Daiana Wischral, Ruben Darío Romero Pelaez, Maria Aparecida de Jesus, Ceci Sales-Campos, Raquel Bombarda Campanha, Thais Demarchi Mendes, Simone Mendonça, Eustáquio Souza Dias, Félix Gonçalves de Siqueira

**Affiliations:** 1 Programa de pós-graduação em Microbiologia Agrícola, Universidade Federal de Lavras (UFLA), Lavras 37200-000, Brazil; 2 Embrapa Agroenergia, Brasília 70770-901, Brazil; 3 Instituto Nacional de Pesquisas da Amazônia (INPA), Manaus 69067-375, Brazil

**Keywords:** *Jatropha*, cottonseed, solid-state fermentation, basidiomycetes, animal nutrition

## Abstract

This work focused on obtaining fermented oil cake (cotton or *Jatropha*) via macrofungi growth with potential characteristics for animal feed formulations, such as the presence of extracellular enzymes, bioactive (ergosterol and antioxidants), and detoxification of antinutritional compounds. The concentration of phorbol esters was reduced by four macrofungi in *Jatropha* seed cake (JSC) to non-toxic levels. At least two macrofungi efficiently degraded free gossypol in cottonseed cake (CSC). Fermentation with *Coriolopsis* sp. INPA1646 and *Tyromyces* sp. INPA1696 resulted in increased ergosterol concentrations, antioxidant activity reduction, and high activity of laccases and proteases. Bromatological analysis indicated high crude protein concentrations, with partial solubilization by fungal proteases. Fermented products from *Coriolopsis* sp. and *Tyromyces* sp. in JSC or CSC can be considered important biological inputs for monogastric and polygastric animal feed.

## 1. Introduction

The sustainability of animal production can be developed by applying alternative lignocellulosic biomass for animal feed. Several physicochemical methods are used to improve the digestibility of these biomasses, such as hydrothermal treatment, ammonia fiber explosion, acid and alkaline treatments. However, the environmental impact of these methods is still a significant concern [1]. Biological treatments, mainly fungal, have gained popularity. Treatments that use white-rot fungi or macrofungi have achieved high levels of efficiency since these fungi selectively degrade lignin, increasing the availability of carbohydrates [2].

Macrofungi are a large group of basidiomycetes with extracellular enzymes that degrade lignin, selectively or not, leaving a white cellulosic and hemicellulosic wood [3]. As far as it is known, these fungi produce secondary compounds such as phenolic compounds, polysaccharides, and enzymes and rarely produce toxic compounds [2]. Fungal delignification occurs by oxidation of lignin through the action of enzymes such as lignin peroxidase (LiP), manganese peroxidase (MnP), and laccase. Non-selective macrofungi tend to degrade cellulose and hemicellulose, while selective fungi mainly secrete hemicellulolytic enzymes, resulting in higher concentrations of cellulose [4]. Therefore, macrofungi with higher selectivity for lignin and lower selectivity for cellulose and hemicelluloses are more efficient in improving the digestibility of biomasses [2].

Cakes or bran that result from the oil extraction of cotton and *Jatropha* seeds have toxic/antinutritional compounds that limit their use. Among these toxic compounds, gossypol is a chemical that causes adverse effects on the growth and reproduction of consuming animals [5]; and phorbol esters can cause acute inflammation and tumors [6]. Despite their toxicity, these co-products are of great interest to the feed industry since they present a high protein content: from 25 to 63% in the cotton cake [5] and from 48 to 64% in *Jatropha curcas* cake [6], or at least 16%, depending on the oil extraction method [7].

Several chemical or biological strategies have been tested to detoxify these residues. However, some of these are not economically viable or only partially solve the contamination problem, allowing the addition only of exceptionally low percentages of the “detoxified” cake in the animal food. Cotton cake can be used in small amounts in the nutrition of ruminants (up to 30%) but is highly toxic for monogastric [5]. *Jatropha* cake is commonly used as an organic fertilizer due to its high content of minerals such as nitrogen, phosphorus, and potassium [8]. 

This work aimed to cultivate macrofungi in solid-state fermentation media containing *Jatropha* seed cake (JSC) and cottonseed cake (CSC), to obtain fermented products with potential characteristics to be used on animal feed formulations, such as the presence of extracellular enzymes, bioactive (ergosterol and antioxidants), and degradation of the antinutritional compounds phorbol esters and gossypol.

## 2. Materials and Methods

### 2.1. Macrofungi

The strains from macrofungi accessed from the Microbiological Collection of the Instituto Nacional de Pesquisas do Amazonas (MC-INPA) were cryopreserved at −80 °C in glycerol 30%. First, fungi were activated in Petri dishes containing commercial Potato Dextrose Agar (PDA), kept in 4 °C and subcultured every three month or when necessary. The ability of fungi to grow on CSC and JSC was evaluated on agar plates. Agar-CSC and Agar-JSC media were prepared with 10% of the crushed dry biomass (JSC or CSC, respectively) and 1.5% of commercial agar, then sterilized for 30 min at 121 °C. The viable macrofungi in PDA were inoculated in plates containing Agar-CSC, Agar-JSC, or PDA (control), and incubated at 28 °C in triplicates. Radial growth of the fungi was measured with a caliper every two days, and those that managed to grow in a medium containing CSC or JSC were selected for solid-state fermentation (SSF).

### 2.2. Solid-State Fermentation (SSF) in JSC and CSC

SSF of macrofungi was carried out in 800 mL glass flasks covered with micropore tape. Substrates were prepared in triplicate with 40 g of JSC or 20 g of CSC (standardization by volume). The moisture level of the biomass was adjusted to 60% with distilled water. Culture flasks were sterilized at 121 °C, JSC for 1 h (phorbol ester is thermostable), and CSC for 20 min (gossypol is thermolabile). Flasks were inoculated with 8 mm mycelial discs (JSC with 10 discs and CSC with 5 discs) and incubated at 28 °C for 15 days or until complete colonization of the biomass. Controls were performed in sterilized flasks containing biomass but without the inoculum of the fungi.

### 2.3. Quantification of Phorbol Esters and Gossypol

The concentrations of the toxic compounds was measured every 3 days until 15 days (phorbol ester in SSF-JSC and gossypol in SSF-CSC). For this, samples were dried at 60 °C for 48 h. Determination of phorbol esters was performed according to Ribeiro et al. [9], with methanol extraction and detection in ultra-performance liquid chromatography (Acquity UPLC H-Class System, Waters, MA, USA) with a PDA detector on Acquity UPLC HSS T3 column (150 mm × 2.1 mm, 1, 8 μm), with pre-column kept at 45 °C, and eluted with quaternary elution gradient, consisting of A: water containing 0.05% trichloroacetic acid; B: Acetonitrile; C: Methanol; and D: Isopropanol; at a flow rate of 0.4 mL/min.. In free gossypol (FG) determination, the dried samples were crushed and homogenized using a bench mill. Extraction and quantification of FG (µg/g) were based on Conceição et al. [10], with acetone extraction and detection in ultra-performance liquid chromatography (Acquity UPLC H-Class System, Waters, Milford, MA, USA) with a PDA detector on Kinetex reverse phase column (100 mm × 2.1 mm × 2.6 μm), with pre-column kept at 35 °C. The PDA detector for acquisition 2D was set at 254 nm, and 3D at 210 to 400 nm. An elution gradient was employed, and the total run time was 14 min.

### 2.4. Determination of the Antioxidant Activity

The best detoxifying fungi were selected according to residual phorbol ester or FG on SSF treatments. Fermentations were repeated with the selected macrofungi to obtain the antioxidant activity profil. The SSF flasks were sacrificed every three days for analysis.

Extraction of total phenolic compounds (TPC) was performed as reported by Asolini et al. [11], with modifications. Each dry sample was weighed (4 g) in Falcon tubes, added 35 mL of 80% (*v*/*v*) ethanol acidified with 0.5% (*v*/*v*) of HCl. Tubes were placed in boiling water for 30 min, the supernatant was separated, and the precipitate was used for another extraction. Second supernatants were removed and placed together with the supernatants from the first extraction. The extracts were centrifuged for 30 min at 6000 rpm and stored at 2 °C in the absence of light.

Total antioxidant activity (TAA) was quantified by the ABTS+ [2,2′-azino-bis(3-ethylbenzothiazoline-6-sulfonic acid)] method and DPPH (2,2-Difenil-1-picril-hidrazila) method. ABTS method was carried out according to Rufino et al. [12]. The ABTS+ radical, generated during the oxidation of ABTS with potassium persulfate, captures hydrogen (electron donor of the antioxidant substance) by the radical, promoting discoloration of the solution. Extract color does not interfere with analysis, and the reagent is soluble in both polar and nonpolar solvents [13].

DPPH method uses a purple and stable free radical, which after receiving the hydrogen atom, is reduced and acquires a yellow color, measured by spectrometry [14]. First, 150 µmol/L DPPH solution, 2000 µmol/L Trolox solution (a synthetic antioxidant analogue to vitamin E), extract dilutions at 1 mg/mL, 0.5 mg/mL and 0.1 mg/mL and a Trolox curve (40–360 µmol/L) were prepared. TAA analyses were performed in wells of a microplate, with 22 µL of each diluted extract or each point of the curve, together with 200 µL of the DPPH solution. Methanol was used as a blank, and absorbance (520 nm) was taken every 15 min, for 1 h, and then every 1 h for more 5 h. The ideal time of activity in the Trolox equivalent was determined. DPPH oxidation inhibition by the time was plotted on a graph to determine the ideal time of antioxidant activity.

### 2.5. Determination of Enzymatic Activities

The best detoxification treatments for JSC and CSC oil cakes were selected, and the fermentation process was repeated for the selected fungi. Every three days, three flasks containing the fermented media were removed for enzymatic activities analysis. Samples from each different treatment (raw cakes, sterilized cakes, and fermented cakes) were homogenized, and 10 g (wet mass) of each were added to 50 mL of cold distilled water (1:5 *m*/*v*). The mixtures were homogenized for 1 h at 200 rpm and 5 °C, then centrifuged for 10 min at 8000 rpm and 4 °C, and supernatant was vacuum filtered. All the crude extracts were stored at 4 °C for the determination of total soluble proteins and enzymatic activities, expressed in U/g of the wet substrate. U was defined as the amount of reducing sugar (μmol) released per minute, and all essays were carried out in triplicate.

Determination of proteolytic activities was done according to the protocol of Charney and Tomarelli [15], with adaptations. Samples were centrifuged at 6000 rpm for 10 min at 4 °C. Tests for proteases present in the extracts were carried out in Elisa microplates, with sodium acetate buffer pH 5 (100 μL) as control. Laccase activity was determined through oxidation of ABTS (Sigma-Aldrich^®^, St. Louis, MO, USA) [16] every 5 s at 420 nm (ε ABTS = 36,000). Total peroxidase activity was determined in 96 well Elisa plates [17].

Determination of FPase activity followed the colorimetric method with DNS according to Xiao et al. [18]. Microassays were carried out to determine endoglucanase (CMCase) and xylanase activities using Carboxy-Methyl-Cellulose (CMC) 2% and xylan beechwood 2%, respectively (Sigma-Aldrich^®^) [19]. The β-glucosidase activity was determined in a 96-well PCR plate, with Cellobiose 15 mM as substrate (Sigma-Aldrich^®^) [20]. Glucose concentrations were quantified using a commercial GOD-POD kit (Bioclin^®^, Belo Horizonte, Brazil) in Elisa plate with absorbance at 505 nm.

### 2.6. Determination of Total Soluble Proteins in the Extracts

The content of total soluble proteins in the crude extracts was determined by the bicinchoninic acid method (BCA) [21] in Elisa plates according to the kit manufacturer’s protocol (Sigma-Aldrich^®^). A dilution curve was obtained through protein concentrations variation to calculate the concentration of total soluble proteins (mg of proteins/mL of extract).

### 2.7. Biomass Bromatological Analysis

Oilseed cakes biomasses from the best detoxification treatments were chemically characterized and compared to the controls: cakes (substrates) without physical (sterilization using autoclave) and/or biological (macrofungus) treatments. CSC and JSC with and without physical and biological treatments were analyzed based on their dry matter content; mineral matter; neutral detergent fiber (NDF); acid detergent fiber (ADF); ether extract; and crude protein. Samples were dried for 48 h with forced ventilation at 25 °C and crushed in a Willey mill (60-mesh). 

Dry matter content was determined after 12 h at 105 °C, and the ash (mineral matter) content was determined after 4 h in a muffle furnace at 600 °C. Determination of the cell-wall components, NDF and ADF were performed according to Van Soest [22]. The ether extract content was determined according to the Am 5-04 method, using an Ankon-type equipment [22]. 

The Kjeldahl method was used to determine the crude protein (CP) content [22]. This same method was used to determine the nitrogen concentration in the samples after aqueous extraction, using a Solvent Accelerated Extractor (Dionex ASE 350) to determine the content of structural proteins. The concentration of soluble proteins was obtained by the difference between crude protein (crude samples) and structural proteins (samples after the extraction process).

### 2.8. Determination of the Structural Carbohydrate Profile (Cellulose, Hemicellulose, and Lignin) in Biomass

Samples were characterized in terms of cellulose, hemicellulose, lignin, and extractive contents according to procedures recommended by the National Renewable Energy Laboratory (NREL, Golden, CO, USA) [23,24,25]. The molar absorption coefficient of the lignin from the JSC and CSC lignocellulosic substrates were determined in the Biomass and Biofuels Chemistry Laboratory at Embrapa Agroenergia as well as other variables related to the biomasses.

### 2.9. Determination of Ergosterol

Ergosterol levels were determined in the selected detoxification treatments for JSC and CSC. Ergosterol from biomasses without macrofungus was also quantified (control). First, dry samples were macerated with liquid nitrogen, 0.5 g of that powder was added to 2 g of KOH, 10 mL of methanol, and 5 mL of ethanol and in a sonicator in the water bath for 30 min at 70 °C. Flasks were kept in the dark and mixture, of 5 mL of distilled water and 10 mL of hexane were added at room temperature. That mixture was then sonicated for 1 min, and the hexane phase (upper) was transferred to rotary evaporator flasks. This extraction was repeated three times. The extracts were evaporated at 40 °C and 300 mbar and resuspended with 2 mL of methanol in a sonicator for 1 min. One mL of each sample was transferred to microtubes, centrifuged, and transferred to vials for injection into ultra-efficient liquid chromatography (Acquity UPLC HClass System, Waters, Milford, MA, USA) with Kinetex column PDA detector 2.6 µm C18 [26].

### 2.10. Determination of the Amino Acids Profile

Amino acid profiles were determined in the best detoxification treatments for JSC and CSC. The same analyzes were carried out for unfermented biomasses (control). Determination of tryptophan content was carried out according to Hagen et al. [27], and those other amino acids and total amino acids were done through HPLC by the CBO group (https://www.grupocbo.com.br, accessed on 6 March 2019) [28].

### 2.11. Statistical Analysis

The results of detoxification, enzymatic activities, and characterization of the treated biomasses were subjected to variance, regression, and correlation analysis by SISVAR [8]. Amin oacids compostion of cakes was determined according to Ahluwalia et al. [29]. For the other tests, the means of the repetitions were compared using the Tukey test, at a 5.0% significance level. 

## 3. Results

### 3.1. Selection of Macrofungi

All viable macrofungi from the collection of the MC-INPA presented homogeneous and vigorous mycelium in PDA petri dishes, with cottony appearance and no spore formation (only vegetative mycelium). Molecular identification confirmed that all fungi belong to the phylum Basidiomycota, with no conclusive at the species level. The growth rate of macrofungi on JSC and CSC-based substrates is shown in Table S1. From the 29 viable macrofungi on PDA, 17 were able to grow in Petri dishes containing only Agar-JSC as a carbon source. The mycelial growth rate varied from 1.92 to 7.37 mm/day for *Trametes elegans* INPA1698 and *Coriolopsis* sp. INPA1646, respectively.

### 3.2. Cake Biodetoxification by Macrofungi

**Figure 1:**
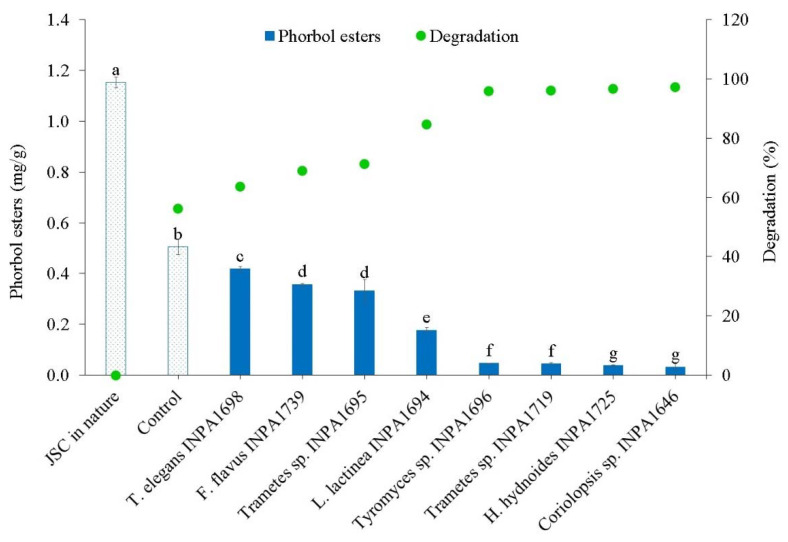
Concentration and degradation of phorbol esters in *Jatropha* seed cake (JSC), after 14 days of SSF by each macrofungi and control (unfermented autoclaved JSC). Different letters indicate that there is a significant difference Tukey test (*p* ≤ 0.05).

Eight macrofungi were able to grow on SSF-JSC and colonized the substrate after 14 days. Figure 1 shows the residual phorbol ester rate on the fermented cakes and the degradation rate of phorbol esters by macrofungi compared to raw cake. 

**Figure 2:**
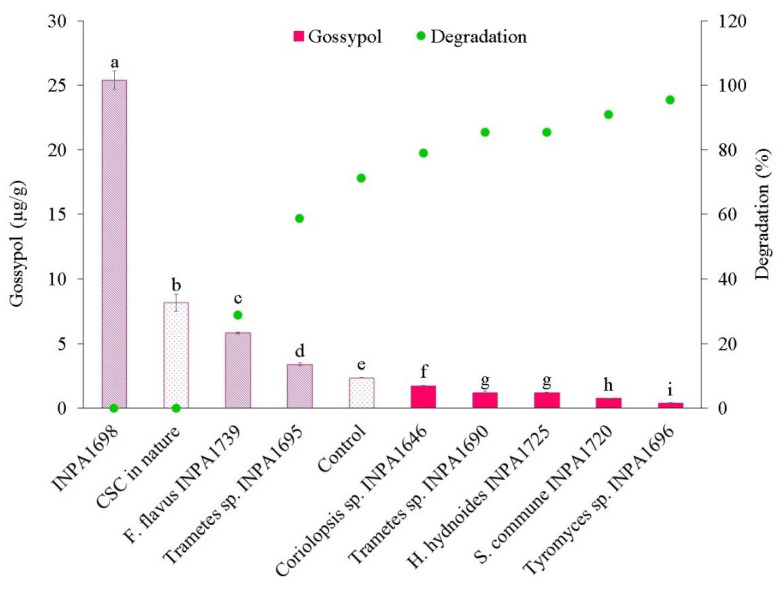
Concentration and degradation of free gossypol on sterilized cotton seed cake (CSC), after SSF by each macrofungi. Different letters indicate that there is a significant difference Tukey test (*p* ≤ 0.05).

Five macrofungi (*Coriolopsis* sp. INPA1646, *Trametes* sp. INPA1695, *Tyromyces* sp. INPA1696, *Schizophyllum commune* INPA1720, and *F. flavus* INPA1739) completely colonized CSC after 14 days. *Trametes* sp. INPA1690 took 20 days for complete colonization, while *T. elegans* INPA1698 and *Hexagonia hydnoides* INPA1725 took 22 days. Figure 2 shows the rate of residual free gossypol on the SSF-CSC and the degradation rate of free gossypol by macrofungi and by sterilization using an autoclave (physical treatment), compared to the raw cake.

### 3.3. Degradation Kinetics of Toxic Compounds

**Figure 3:**
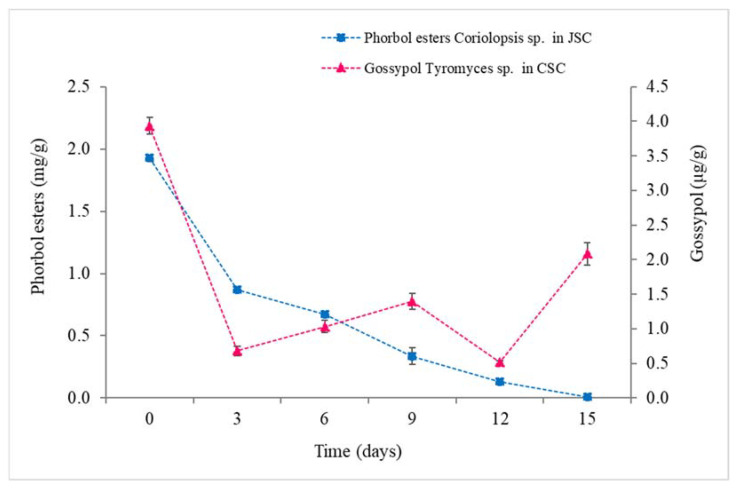
The concentration of phorbol esters in SSF-JSC by *Coriolopsis* sp. and of gossypol in SSF-CSC by *Tyromyces* sp., every 3 days of cultivation.

Fermentation of the cakes was repeated with the selected macrofungi, *Coriolopsis* sp. INPA1646 for JSC detoxification and *Tyromyces* sp. INPA 1696 for CSC detoxification (Figure S1). These fungi were selected for their efficiency in degrading phorbol esters (Figure 1) and free gossypol (Figure 2). Biological triplicates were carried out for 15 days to determine the levels of phorbol esters and free gossypol on SSF-JSC and SSF-CSC, respectively (Figure 3). It is noteworthy that macrofungi’s solid-state fermentation processes of agro-industrial residues generally occur for longer periods, as previously reported in the literature.

### 3.4. Determination of Antioxidant Activity

**Figure 4:**
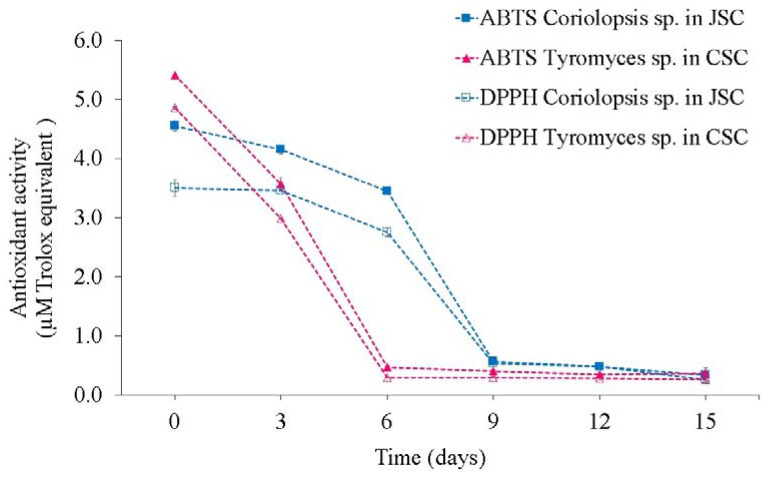
Determination of antioxidant activity, through ABTS and DPPH methods, for SSF-JSC and SSF-CSC using macrofungi *Coriolopsis* sp. and *Tyromyces* sp., respectively.

Antioxidant activity of SSF-JSC and SSF-CSC, using *Coriolopsis* sp. and *Tyromyces* sp., respectively, were evaluated (Figure 4). Both determination methods resulted in identical activity profiles, with slightly higher activities by the ATBS method.

### 3.5. Determination of Total Soluble Proteins and Enzymatic Activity

The extract that provided the highest concentration of total soluble proteins was the JSC fermented for 15 days by *Coriolopsis* sp. (Figure S2). The BCA method quantifies the fungal proteins released in the medium, including the enzymes. However, the aqueous extraction also removes the soluble vegetable proteins. Hence, the high content of total soluble proteins does not necessarily indicate a high concentration of secreted fungal enzymes.

Throughout fermentation, levels of total soluble proteins on JSC were higher than in CSC. The highest concentration (4 mg/mL) was reached on the 15th day of fermentation. A fraction of fungal proteins produced during substrate colonization (proteolytic enzymes) presumably is also responsible for the solubilization of vegetable proteins observed in the bromatological characterization. 

**Figure 5:**
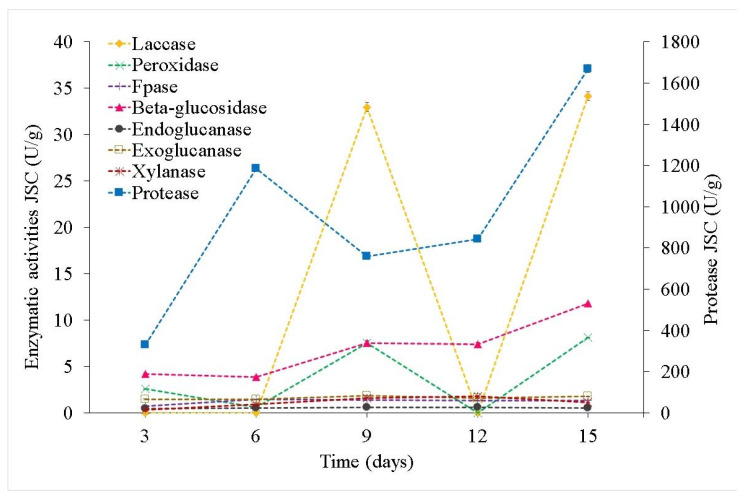
Activity kinetics of soluble enzyme activities (laccase, peroxidase, Fpase, β-glucosidase, endoglucanase, exoglucanase, xylanase, and protease) in the crude extracts of SSF-JSC with *Coriolopsis* sp.

**Figure 6:**
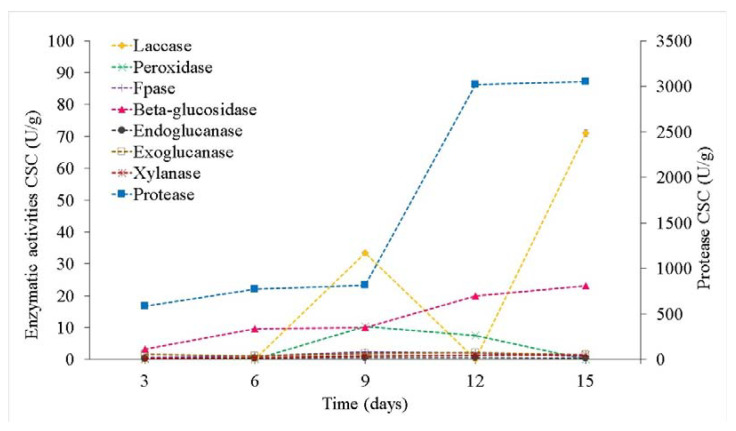
Activity kinetics of soluble enzyme activities (laccase, peroxidase, Fpase, β-glucosidase, endoglucanase, exoglucanase, xylanase, and protease) in the crude extracts of SSF-CSC with *Tyromices* sp.

The difference in the protease activities for the two crude extracts from SSF-CSC and SSF-JSC was clear (Figure 5 and Figure 6), *Tyromyces* sp. (CSC) provided better results than *Coriolopsis* sp. (JSC). The highest protease production occurred on the 15th day of SSF-CSC by *Tyromyces* sp., with an activity of 3051 U/g (Figure S3). For *Coriolopsis* sp. on SSF-JSC the maximum protease production was 1670 U/g, reached on the last day of fermentation. 

The maximum laccase activity was observed for SSF-CSC using *Tyromyces* sp. INPA1696, for 15 days, with an activity of 71 U/g (Figure 6). This is 2-fold the highest activity observed using *Coriolopsis* sp. on SSF-JSC, which was only 34 U/g (Figure 5). 

The highest activity of total peroxidases was observed on the 9th day of SSF-CSC using *Tyromyces* sp., with 10 U/g, while until the 6th day no activity was observed (Figure 6). And on the 12th day of fermentation, the activity was 7.5 U/g, similar to the 9th day of SSF-JSC using *Coriolopsis* sp. (Figure 5 and Figure 6). 

*Tyromyces* sp. showed higher total cellulase activity in SSF-CSC (FPase) than Coriolopsis sp. in SSF-JSC. With maximum production of 2.3 U/g on the 9th day of fermentation (Figure 6).

β-glucosidases from *Tyromyces* sp. in CSC presented the highest activity on the 12th and 15th days of fermentation, with 20 and 23 U/g, respectively (Figure 6). However, β-glucosidases activity by *Coriolopsis* sp. in JSC remained at basal levels, with a maximum of 12 U/g on the 15th day.

Exoglucanases cleave bonds at the ends of the cellulose chain. These were the enzymes that presented the lowest activity during CSC fermentation. In both fermentations, endo and exoglucanase activities remained similar and stable, varying between 0.3 and 0.6 U/g of endoglucanase and from 1.4 to 1.8 U/g of exoglucanase with a maximum activity of 2.2 U/g on the 12th day of SSF-CSC (Figure 5 and Figure 6). The low solubility of avicel, used as a substrate here, resulted in high standard deviations. Hence, it is not possible to affirm a statistically significant difference in the observed activity levels.

*Coriolopsis* sp. showed increasing xylanase activities until the 12th day of JSC fermentation, reaching 1.8 U/g with a decrease on the 15th day (Figure 5). *Tyromyces* sp. extract activities decreased from the 3rd to 6th day, after which an increase was observed, lasting until the end of the fermentation with a maximum of 1.5 U/g on the 15th day (Figure 6).

### 3.6. Bromatological Analysis of the Fermented SSF-JSC and SSF-CSC

The 14-day fermented SSF-JSC and SSF-CSC by *Coriolopsis* sp. and *Tyromyces* sp., respectively, were characterized in terms of bromatological analysis and concentration levels of cellulose, hemicellulose, and lignin (NREL). SSF-JSC and SSF-CSC presented a reduction of 10% reduction in their dry mass content (Table 1).

### 3.7. Structural Carbohydrates (Cellulose, Hemicellulose, and Lignin) by NREL Analysis Methods

Among the sugars investigated, only arabinan and galactan had their concentration reduced on SSF-JSC. CSC samples do not contain mannan and ramnan, only glucans (cellulose), and it does not have concentrations reduced (Table 2).

Based on sugar concentration after biomass deconstruction is possible to determine the contents of cellulose (glucan) and hemicellulose (other sugars). Besides, via NREL method it is possible to determine the concentration of lignin in the biomass (Table 2). 

NREL analyses determined structural inorganic compounds for both biomasses that decrease significantly at the end of fermentation, which indicates the solubilization of these by fungi during their growth and increases the availability of these compounds in the fermented biomasses. For JSC, levels of structural inorganics compounds decreased from 3.57 ± 0.08% raw cake, to 3.23 ± 0.07% after autoclaving and to 1.91 ± 0.06% after fermentation by *Coriolopsis* sp. In CSC, levels were reduced from 2.06 ± 0.10% in raw cake and 2.18 ± 0.04% in autoclaved (without a statistical difference) to 1.34 ± 0.02% after the growth of *Tyromyces* sp. 

### 3.8. Determination of Ergosterol

At the end of the 14 days of cultivation, the concentration of this sterol increased 10 times, reaching 201.88 ± 8.8 µg/g in JSC fermented. CSC without fungus did not show detectable ergosterol, however, the cake fermented for 14 days by *Tyromyces* sp. presented 147.12 ± 20.37 µg/g of ergosterol. It was observed that unfermented JSC has ergosterol in a concentration of 22.78 ± 3.93 µg/g of cake ergosterol.

### 3.9. Composition of Amino Acids

Vegetable proteins are converted into fungal proteins through fermentation. The total amount of protein, in general, does not change since nitrogen in the substrate is not lost during fermentation, nor is nitrogen in the environment fixed to the substrate by the fungus. However, what is observed is an increase in nitrogen content due to the loss of carbon by fungus respiration. As the analysis of crude proteins is done by quantifying the total nitrogen of the biomass, the concentration of crude proteins may increase, even if the concentration of amino acids has decreased (Table 3). 

## 4. Discussion

### 4.1. Selection of Macrofungi

*Flavodon flavus* INPA1739 and *Coriolopsis* sp. INPA1646 showed the highest growth rate on the Agar-CSC and Agar-JSC plates, respectively. The lowest growth rate on Agar-CSC and Agar-JSC was observed for *T. elegans* INPA1698. However, some fungi that grew on the plates with Agar-JSC or Agar-CSC did not grow on SSF-JSC or SSF-CSC. *Coriolopsis* sp. INPA1646 presented the highest growth rate in glass flasks SSF-CSC and SSF-JSC. The lowest growth rate was observed for *Trametes* sp. INPA 1719 on SSF-JSC and for *T. elegans* INPA 1698 on SSF-CSC.

### 4.2. Cake Biodetoxification by Macrofungi

Raw JSC presented 1.15 mg of phorbol ester per gram of cake, similar to the results of Bose and Keharia [30] of 1.07 mg/g. The *Jatropha* cake is considered non-toxic when phorbol esters range from 0–0.09 mg/g [31]. The best degradation result of this work was found with 14 days of growth of *Coriolopsis* sp. INPA1646, with only 0.023 mg/g residual phorbol ester.

The degradation of phorbol esters through SSF-JSC by basidiomycetes is widely discussed in the literature (Table S2) and the detoxification process of JSC by *P. ostreatus* is very well established. However, Kasuya et al. [32] observed only 85% degradation of phorbol esters in 30 days of SSF-JSC by *P. ostreatus*, lower than the 97% and 91% observed by Barros et al. [33] using *Phlebia rufa* and *Bjerkandera adusta*, respectively, and the 97.22% found in this work with only 14 days of growth of *Coriolopsis* sp. (Figure 1).

*Trametes elegans* INPA1698, *Flavodon flavus* INPA1739 and *Trametes* sp. INPA1695 causes an increase in FG concentration in the fermented cottonseed cake instead of degrading IT (Figure 2). That was probably due to the release of gossypol bound to the protein by the action of these fungi.

Degradation of free gossypol in CSC is not exceedingly difficult to occur due to its instability. Furthermore, degradation of this substance by fungal fermentation has been reported in the literature, with reductions of gossypol up to 65.2%, by *Fusarium thapsinum* [34]. No reports were found regarding the degradation of free gossypol by *Tyromyces*, here the degradation of free gossypol by this fungus (INPA169) was 95.34%, with a residual rate of only 0.38 µg/g.

According to Rajarathnam et al. [35], *Pleurotus florida* could detoxify 100% of gossypol after 5 days, in a medium containing 100 µg/g of gossypol. Treatment of CSC using other microorganisms, such as *Candida tropicalis*, *Saccharomyces cerevisiae*, *Aspergillus niger*, and *Aspergillus terricola* could also significantly degrade the free gossypol in the cake (up to 88.51%) after 15 days of incubation [36].

### 4.3. Degradation Kinetics of Toxic Compounds

CSC autoclaving for 20 min degrades up to 89.4% of the free gossypol in the cake. However, during fermentation by *Tyromyces* sp., levels of free gossypol (FG) vary, increasing and decreasing its concentration, indicating that the fungus may degrade but also release bounded gossypol (BG) during fermentation (Figure 3). Despite that, the degradation process continues to occur due to the enzymatic activities involved in the process, as demonstrated in previous works in literature which longer fermentation processes result in complete degradation of gossypol in CSC. That corroborates to Soares-Neto [37], which indicates a series of genes and proteins from *P. lecomtei* BRM 044603 that could be associated with the CSC detoxification process. 

The highest percentage of phorbol esters degradation in SSF-JSC was 99.51% and occurred on the 15th day of fermentation, and the degradation was 93.37 after 12 days. This result was higher than the experiments carried out in Sharath et al. [38], where the maximum phorbol esters degradation was 75% after 12 days of fermentation of JSC by *Cunninghamella echinulata*. 

### 4.4. Determination of Antioxidant Activity

In general, antioxidant activity declines throughout SSF, probably due to the action of oxidative enzymes, such as laccases and peroxidases, or by the action of other fungal bioactive secreted during fermentation and products formed by enzymatic reactions [39]. Extracellular oxidative enzymes are involved in the depolymerization of lignin, caused mainly by the generation of free radicals by oxidases and peroxidases [40]. 

Cakes, for themselves, present high antioxidant activity [7]. The high antioxidant activity of JSC is attributed to high concentrations of phenolic compounds [41] and, in CSC due to the presence of peptides with antioxidant activity, which reduces catechin oxidation [42] and the presence of secondary metabolites such as gossypol [43]. Guimarães et al. [41] observed a reduction of 97.8% of the antioxidant activity in JSC and CSC after 15 days of macrofungi fermentation. The antioxidant activity in ABTS and DPPH of unfermented cakes were higher than those observed in this work, for JSC was 147.9 and 109.4 uM of Trolox equivalent per mg of extract, respectively, and for CSC was 122.1 and 53.5 uM Trolox equivalent, respectivetly [41].

Kumar et al. [43] observed that the antioxidant activity in CSC is directly proportional to the concentration of gossypol, which acts as an antioxidant, and reported an average activity of 0.2% reduction of free radicals. However, macrofungi are known to produce substances with antioxidant activity, such as phenolic compounds, vitamins, flavonoids, and tocopherols [3,26]. Fermentation of biomasses by other fungi, such as *A. oryzae*, in cottonseed meal, for 48 h increased the antioxidant activity of the bran, and its addition to the tilapia diet increases the antioxidant activity in the diet and liver of fish [44]. But, under the studied conditions, reducing the natural antioxidant activity of JSC and CSC cakes by fungal enzymes is more significant than producing any fungal compounds with antioxidant activity.

### 4.5. Determination of Total Soluble Proteins and Enzymatic Activity

Proteases can be produced and released by a wide variety of microorganisms, intracellular or extracellular, being linked or not to the membrane. Approximately 40% of the commercial proteases are of microbial origin, which is preferred to those of animal and plant origins due to their lower production cost and more advantageous characteristics for biotechnological applications [45].

Yang et al. [46] suggested that some proteases are closely linked to the degradation of free gossypol. Then, probably because of this, the peak of protease activity for CSC was observed on the 12th day (Figure 6), the same day on which a significant reduction in the levels of free gossypol (Figure 3) was observed, as well as a lower concentration of soluble proteins during cultivation (Figure S2). These might have been metabolized by macrofungus in the form of amino acids or peptides. 

Laccase production by *Coriolopsis caperata* RCK2011 under solid-state fermentation of wheat bran was optimized by altering fermentation conditions such as temperature, pH, inoculum size, and concentration of corn liquor, KH_2_PO_4_, biotin, xylidine (an inducer), and copper, achieved a 58% increase in laccase production, reaching 1623.55 U in five days [47]. Other *Coriolopsis* species, such as *C. gallica* [48], *C. rigida* [49], and *C. polyzina* [50] are also known to produce laccase. Similarly, some *Tyromyces* species such as *T. xuchilensis* [51], *T. chioneus* and *T. pubescens* [52] have also been reported as ligninase producers.

Ncube et al. [53] optimized the production of cellulases and xylanase by *A. niger* in SSF-JSC by altering factors such as cake supplementation with 10% common thatch grass (*Hyparrhenia* sp.) and ammonium chloride, nitrogen sources, temperature, and pH. It was observed that each enzyme is favored by specific parameters and under different conditions of temperature and pH, being that the maximum cellulase activity observed was 3974 U/g and 6087 U/g of xylanase.

### 4.6. Bromatological Analysis of the Fermented SSF-JSC and SSF-CSC

Results from Table 1 could be explained by the loss of carbon in the form of CO_2_ during fungal respiration, also explain the increase in the levels of other components, such as ashes, which increased at least 0.43% on SSF-JSC. It might have occurred a proportional increase in the content of inorganic compounds to the detriment of organic matter lost during fermentation. On SSF-CSC, all the other levels, except for ether extract (EE), increased with the growth of *Tyromyces* sp. 

Neutral detergent fiber (NDF) content on SSF-JSC and SSF-CSC presented a slight increase, probably because of lignin concentration due to degradation of cellulose and other less complex carbohydrates. Biomass structural carbohydrates with low digestibility interfere negatively with the animal’s diet.

NDF is the nutritional fraction corresponding to the sum of hemicellulose, cellulose, and lignin. Hemicellulose is highly digestible; cellulose presents variable digestibility, and lignin is practically indigestible. Although there is no reduction in the concentration of lignin in the fermented biomass, the growth of the fungus may have modified the structure of this polymer, resulting in less recalcitrant lignin. ADF is the sum of cellulose and lignin, and the higher its content means higher levels of lignin, which implies less digestibility. In conventional bromatological analyzes, hemicellulose is calculated by the difference between ADF and NDF, and cellulose by the difference between lignin and ADF [54]. Wang et al. [55] reported a reduction of 25.79% to ADF, 30.74% to NDF, 26.62% to cellulose, and 31.44% to hemicellulose after 21 days of corn straw fermentation by different species of *Pleurotus.*

In terms of nutrition, ADF allows the calculation of total digestible nutrients (TDN), which are directly related to digestibility, which corresponds to the energy from the food. For such, the following formula should be applied: % TDN = 87.84 − (0.70 x % FDA). Then the values for TDN were 59.46% for raw JSC, 63.16% for autoclaved JSC, and 56.19% after fungus growth. 

Ether extract (EE) refers to the contents of oils and fats in the analyzed samples. Some microorganisms, including some known macrofungi (Basidiomycetes), use lipids as an energy source during growth through the release of lipases on the substrate [7], resulting in a reduction in the levels of ether extract by the end of cultivation, as observed for both SSF-JSC and SSF-CSC samples (Table 1). Wang et al. [55] observed an increase in the concentration of EE after 21 days of *Pleurotus* species growth in corn straw. That occurs because the JSC fat varies according to the origin of the seeds and the efficiency of oil extraction. *Coriolopsis* sp. and *Tyromyces* sp. used the ether extracts present in JSC and CSC, respectively.

Despite fungi (Basidiomycetes) cannot fix nitrogen, the fermentation of plant substrates by these macrofungi has increased the levels of proteins and amino acids [36], probably due to the consumption of other nutrients in the substrate, such as carbohydrates and lipids. The loss of carbon, in the form of CO_2_, via fungal respiration, increases the nitrogen concentration in the substrate, which increases Crude protein because of the concentration caused by that carbon loss [56]. Results show there was no significant nitrogen concentration on the SSF-JSC, and levels of CP did not present significant changes. Zhang et al. [36] demonstrated an apparent increase in the levels of crude protein in CSC colonized by the fungi *Candida capsuligena*, *C. tropicalis*, *S. cerevisiae*, *Aspergillus oryzae*, and *A. niger*. Wang et al. [55] also observed an increase in CP concentration of up to 31.66% in corn straw 21 days after the growth of different species of *Pleurotus*. 

The fermentation process seems to solubilize part of crude protein, although it does not cause an increase in levels, its nitrogen becomes more available. This can be seen by the reduction in concentrations of a structural protein and the increase in soluble proteins (Table 1). Almost none of the protein present in CSC was soluble before fermentation. During the growth of *Tyromyces* sp. in the cake, solubilization of part of the structural protein present in the biomass occurred, representing an advantage of the fermentation process. This was probably due to the production and release of proteolytic enzymes, which presented significant activity on SSF-CSC when compared to the fermentation of JSC (Figure 6).

Crude protein levels in raw JSC were 27.35%, similar to the levels reported in the literature, which vary from 28.87% to 37.82% [56]. Some studies indicate an increase in concentrations of crude protein on JSC around 8%, when treated with *Absidia spinosa* and *Mucor rouxii* [57], or a reduction when treated with *Trichoderma longibrachiatum*, *Trichoderma harzianum*, and *A. niger* [58].

### 4.7. Structural Carbohydrates (Cellulose, Hemicellulose, and Lignin) by NREL Analysis Methods

According to literature, carbohydrates in mushrooms include β-glucans, D-galactose, D-mannose, D-xylose, L-fructose, L- (or D) -arabinose, xylose, fructose, mannose, glucose, sucrose, and mannose in the mycelium of *P. ostreatus*, *P. eryngii*, *P. tuberregium*, *G. lucidum*, *P. baumii*, *A. bisporus*, *F. velutipes*, *L. edodes*, *A. blazei*, *S. crispa*, and *I. obliquus*, among others [3]. 

Degradation of hemicellulose and cellulose produces free fermentable sugars, which stand out as the focus of research concerning alternative fuel sources (second-generation ethanol), indicating other potential applications of solid-state cultivation of basidiomycetes in lignocellulosic biomasses [1,54]. In the current work, as there was no observed reduction in lignin levels for any biomass, it was possible to infer that the isolates *Coriolopsis* sp. and *Tyromyces* sp. did not degrade lignin throughout JSC and CSC fermentations. 

### 4.8. Determination of Ergosterol

Ergosterol in unfermented JSC can be explained since the presence of an endophytic fungus is considered a natural contaminant of this oilseed. Although JSC was sterilized before inoculation with the macrofungi tested, ergosterol from the dead fungi could still be detected by the method.

### 4.9. Composition of Amino Acids

Nayan et al. [59] reported an increase in arginine content of 56% and lysine content of 15% after colonization of wheat straw by basidiomycetes, while here there was a small reduction in both amino acids. The amino acid profile was altered because plant and fungal proteins can have different amino acid profiles. As seen in Table 3, the most common amino acid in the evaluated biomasses is glutamic acid, followed by arginine. Non-essential amino acids are those synthesized by the body itself and are involved in many metabolic functions, being glutamate a precursor to glutamine, which is required by several cells [60].

Lim [44] observed higher concentrations of all amino acids in cottonseed meal than found here for CSC. They obtained little change in the concentrations after fermentation by *A. oryzea* at 48 h, increased concentrations of arginine, isoleucine, methionine, phenylalanine, tryptophan, cysteine and glycine; and reduction of histidine, leucine, lysine, threonine, valine, alanine, serine, aspartic acid, and glutamic acid. In this work, CSC fermentation for 15 days reduced all amino acids, except for threonine. The most abundant amino acid in cottonseed meal samples was glutamic acid (10.9%), as well as in CSC (3.4%).

The amino acid concentrations of JSC reported by Devappa and Swamylingappa [39] were higher than those found here, and the amino acid in the highest concentration was also glutamic acid with 18.5%. These works show that amino acid concentrations in oilseed cakes can vary greatly between batches of different origins. Amino acid concentrations in JSC protein concentrates are also higher than those found here, except histidine, and the most abundant amino acid in these concentrates is also glutamic acid [59].

Peter et al. [61] established the optimal amino acid concentrations in corn-based feed for young chicks, and the main amino acid is L-glutamine, with 10.9%, followed by L-tryptophan (1.19%), L-Cysteine (0.81%), L-Threonine (0.39%), L-Valine (0.36%), L-Isoleucine (0.28%), L-Cysteine (0.19), DL-Methionine (0.17%) and L-histidine (0.14%). As for mammals, they all require the core of nine essential amino acids (Table 4), but they can also respond to dietary arginine and proline during the early phases of rapid growth [62].

The net amino acid requirements for weight gain in uncastrated Nelores vary about the live weight (LW) of the animal and the method used to calculate, which were determined by Silva et al. [63]. By the factorial method, the requirements decreased with the increase of the animal’s live weight, as a consequence of the greater accumulation of fat. If calculated by the method of equations, the requirements of lysine, histidine, phenylalanine, tryptophan, leucine, isoleucine, asparagine, glutamine, tyrosine and cystein increased with increasing of the animal’s live weight, while those of valine, arginine, serine, glycine and alanine decreased [63].

## 5. Conclusions

The results showed that 64% of viable macrofungi from the Microbiological Collection–MC INPA grew in Petri dishes with JSC or CSC as the only substrate. Among these fungi, 62.5% grew in SSF flasks with those biomasses. Four fungi (*Tyromyces* sp. INPA1696, *Trametes* sp. INPA1719, *H. hydnoides* INPA1725 and *Coriolopsis* sp. INPA1646) grew in JSC and managed to detoxify this substrate after 14 days of fermentation, with a degradation rate of phorbol esters up to 97.22%. In CSC, the degradation rate of free gossypol reached 95.34%; however, the release of bound gossypol by some fungi made levels of free gossypol increase. Antioxidant activity of JSC and CSC cakes decreased on the 6th and 9th days of fermentation by *Coriolopsis* sp. and *Tyromices* sp., respectively. *Tyromices* sp. was an excellent producer of proteolytic and cellulolytic enzymes in CSC. Both fermented biomasses presented potential reduction of toxic/antinutritional factors and are rich in proteins, amino acids, enzymes, and ergosterol, which are promptly available for animal feed. 

## Figures and Tables

**Table 1:** Bromatological analysis of JSC and CSC before and after fermentation (SSF) by *Coriolopsis* sp. and *Tyromices* sp., respectively (% ± SD).

Samples	JSC Raw	JSC (Autoclaved)	SSF-JSC *Coriolopsis* sp. INPA1646	CSC Raw	CSC (Autoclaved)	SSF-CSC *Tyromices* sp. INPA1696
DM	97.12 ± 0.16 ^a^	97.20 ± 0.05 ^a^	87.18 ± 0.12 ^d^	93.73 ± 0.47 ^c^	95.41 ± 0.47 ^b^	82.99 ± 0.09 ^e^
Ashes	4.66 ± 0.05 ^d^	4.52 ± 0.10 ^d^	5.33 ± 0.19 ^b^	5.47 ± 0.05 ^b^	5.00 ± 0.08 ^c^	6.80 ± 0.15 ^a^
CP	27.35 ± 1.12 ^a^	28.59 ± 1.20 ^a^	28.27 ± 1.04 ^a^	21.19 ± 1.58 ^b^	20.73 ± 0.8 ^b^	23.58 ± 2.24 ^b^
EP	19.17 ± 0.69 ^b^	18.82 ± 0.11 ^b^	16.42 ± 0.66 ^d^	20.96 ± 2.36 ^a^	20.94 ± 1.06 ^a^	18.03 ± 0.87 ^c^
SP	8.18 ± 0.48 ^c^	9.77 ± 0.85 ^b^	11.85 ± 0.97 ^a^	0.23 ± 0.05 ^d^	0.21 ± 0.06 ^d^	11.50 ± 1.14 ^a^
NDF	52.74 ± 1.24 ^d^	53.64 ± 0.55 ^c^	56.89 ± 0.83 ^a^	51.10 ± 1.99 ^d^	54.55 ± 1.65 ^b^	54.03 ± 1.96 ^b^
ADF	40.54 ± 0.76 ^b^	35.25 ± 0.44 ^d^	45.22 ± 0.62 ^a^	34.89 ± 0.56 ^d^	36.96 ± 0.32 ^c^	40.29 ± 1.47 ^b^
CF	37.54 ± 1.34 ^a^	34.00 ± 0.32 ^b^	38.50 ± 0.78 ^a^	28.80 ± 2.19 ^cd^	28.82 ± 0.47 ^d^	30.90 ± 0.66 ^c^
Lig	23.36 ± 0.41 ^b^	19.21 ± 0.45 ^c^	28.72 ± 0.55 ^a^	9.19 ± 0.51 ^b^	10.09 ± 0.35 ^b^	14.27 ± 0.35 ^a^
EE	12.62 ± 0.07 ^a^	13.16 ± 0.46 ^a^	5.07 ± 0.42 ^b^	3.53 ± 0.19 ^c^	0.48 ± 0.12 ^e^	0.84 ± 0.02 ^d^

Legend: DM = dry mass; CP = crude protein; EP = Estructural protein; SP = Soluble protein; NDF = neutral detergent fiber; ADF = acid detergent fiber; CF = crude fiber; Lig = lignin; EE = ether extract. Letters next to the values correspond to the comparisons of the averages on the same line and not between one line and another (Tukey test *p* ≤ 0.05).

**Table 2:** Concentrations of sugars, cellulose, hemicellulose and lignin in JSC and CSC before and after fermentation (SSF) by *Coriolopsis* sp. and *Tyromyces* sp., respectively (% ± SD).

Component	JSC Raw	JSC Autoclaved	SSF-JSC *Coriolopsis* sp. INPA1646	CSC Raw	CSC Autoclaved	SSF-CSC *Tyromyces* sp. INPA1696
Glucan	17.67 ± 0.12 ^e^	19.99 ± 0.54 ^d^	19.87 ± 0.48 ^d^	24.65 ± 0.61 ^c^	26.37 ± 1.18 ^b^	38.31 ± 1.67 ^a^
Xylan	11.62 ± 0.37 ^a^	11.84 ± 0.35 ^a^	11.64 ± 0.27 ^a^	10.32 ± 0.79 ^b^	10.18 ± 3.54 ^b^	9.95 ± 0.94 ^b^
Mannan	1.61 ± 0.09 ^a^	1.53 ± 0.01 ^b^	1.52 ± 0.05 ^b^	0.0	0.0	0.0
Arabinan	1.64 ± 0.16 ^b^	1.24 ± 0.04 ^c^	0.31 ± 0.01 ^e^	3.11 ± 0.05 ^a^	1.09 ± 0.32 ^c^	0.44 ± 0.06 ^d^
Galactan	1.00 ± 0.06 ^a^	0.86 ± 0.04 ^b^	0.36 ± 0.02 ^e^	1.01 ± 0.03 ^a^	0.77 ± 0.03 ^c^	0.40 ± 0.02 ^d^
Ramnan	0.83 ± 0.08 ^b^	0.58 ± 0.07 ^c^	1.38 ± 0.11 ^a^	0.0	0.0	0.0
Cellulose	17.67 ± 0.12 ^d^	19.99 ± 0.54 ^c^	19.87 ± 0.47 ^c^	24.65 ± 0.61 ^b^	26.37 ± 1.18 ^b^	38.31 ± 1.67 ^a^
Hemicellulose	16.70 ± 0.75 ^a^	16.06 ± 0.52 ^a^	15.19 ± 0.46 ^b^	14.96 ± 0.87 ^b^	14.59 ± 3.89 ^bc^	11.83 ± 1.02 ^c^
Lignin	22.02 ± 0.60 ^d^	21.61 ± 0.32 ^e^	25.68 ± 0.50 ^c^	34.93 ± 1.03 ^a^	43.93 ± 3.58 ^a^	39.12 ± 1.96 ^b^

Letters next to the values correspond to the comparisons of the averages on the same line and not between one line and another (Tukey test *p* ≤ 0.05).

**Table 3:** Amino acid composition in *Jatropha* seed (JSC) and cottonseed (CSC) cakes before and after fermentation using *Coriolopsis* sp. and *Tyromyces* sp., respectively, for 14 days.

Amino Acids	JSC Raw	JSC Autoclaved	SSF-JSC *Coriolopsis* sp. INPA1646	CSC Raw	CSC Autoclaved	SSF-CSC *Tyromyces* sp. INPA1696
Essential						
Histidine	0.58%	0.48%	0.46%	0.70%	0.67%	0.55%
Isoleucine	0.97%	0.86%	0.73%	0.84%	0.83%	0.79%
Leucine	1.63%	1.47%	1.23%	1.52%	1.52%	1.25%
Lysine	0.77%	0.55%	0.63%	0.99%	0.96%	0.91%
Methionine	0.28%	0.27%	0.22%	0.36%	0.36%	0.31%
Phenylalanine	1.01%	0.90%	0.76%	1.35%	1.35%	0.98%
Threonin	0.83%	0.75%	0.69%	0.82%	0.83%	0.85%
Tryptophan	0.11%	0.12%	0.11%	0.13%	0.11%	0.11%
Valine	1.14%	1.03%	0.88%	1.07%	1.11%	1.03%
Arginine	2.51%	2.09%	1.38%	2.78%	2.70%	2.01%
Nonessential						
Alanine	1.05%	0.95%	0.86%	0.97%	0.98%	0.96%
Aspartic acid	2.06%	1.80%	1.82%	2.26%	2.19%	2.14%
Cystine	0.37%	0.34%	0.32%	0.40%	0.40%	0.33%
Glutamic acid	3.40%	3.08%	2.29%	5.02%	4.89%	3.73%
Glycine	1.01%	0.90%	0.89%	1.08%	1.09%	0.98%
Proline	0.94%	0.84%	0.73%	0.94%	0.93%	0.88%
Tyrosine	0.65%	0.58%	0.44%	0.76%	0.74%	0.62%
Serine	1.12%	1.01%	0.94%	1.17%	1.13%	1.03%
Taurine	<0.01	<0.01	<0.01	<0.01	<0.01	<0.01
Total amino acids	20.44%	18.02%	15.37%	23.16%	22.78%	19.45%
Crude protein	22.98%	22.56%	26.63%	28.43%	29.13%	30.89%

Raw: cake from pressing the seeds, dried at 60 °C for 24 h and crushed in a bench mill. Control: cake autoclaved with 60% humidity and incubated for 14 days at 28 °C, without fungus, and then dried at 60 °C for 24 h and crushed in a bench mill. Fermented: cake autoclaved with 60% humidity, incubated for 14 days at 28 °C with the macrofungi, dried at 60 °C for 24 h, and ground in a bench mill.

**Table 4:** Nutritional classification of amino acids [62].

Common Core	Additional Species-Related Requirements	Conditionally Non-Essential	Non-Essential
Lysine	Arginine (cats, poultry, fish)	Cyst(e)ine	Glutamate
Histidine	Taurine (cats)	Tyrosine	Glutamine
Leucine		Arginine	Glycine
Isoleucine		Proline	Serine
Valine			Alanine
Methionine			Aspartate
Threonine			Asparagine
Tryptophan			
Phenylalanine			

## Data Availability

Not applicable.

## Supplementary Materials

The following supporting information can be downloaded at: https://www.mdpi.com/article/10.3390/microorganisms10081670/s1, Figure S1: Pictures of the glass flasks containing the fermented cakes after 3, 6, 9, 12 and 15 days of incubation at 28 °C, to illustrate the level of colonization of JSC and CSC by macrofungi *Coriolopsis* sp. and *Tyromices* sp., respectively; Figure S2: Total soluble protein kinetics in the crude extracts of SSF-JSC and SSF-CSC using *Coriolopsis* sp. and *Tyromices* sp., respectively; Figure S3: Total enzyme activities in the crude extracts of SSF-CSC and SSF-JSC using macrofungi *Tyromices* sp. and *Coriolopsis* sp., respectively, after 15 days of fermentation; Table S1: Growth rate (mm/day) of macrofungi on Agar-JSC plates, SSF-JSC flasks and CSC-Agar and SSF-CSC flasks; Table S2: Literature results for degradation of phorbol esters in JSC, via solid-state fermentation by macrofungi.
