# Supplementary material for: Bioactives and Extracellular Enzymes Obtained from Fermented Macrofungi Cultivated in Cotton and Jatropha Seed Cakes
Source: Microorganisms. 2022 Aug 19;10(8):1670. doi: 10.3390/microorganisms10081670 (PMC9413263; doi:10.3390/microorganisms10081670)
Supplement: Supplementary file 1 [file microorganisms-10-01670-s001.zip › microorganisms-1844542-supplementary.pdf]

## Supplementary material

**Table S1.** Growth rate (mm/day) of macrofungi on Agar-JSC plates, SSF-JSC flasks and CSC-Agar and SSF-CSC flasks

| Fungi | | Growth-rate (mm/day) ± SD | | | |
|---|---|---|---|---|---|
| **Code** | **Name** | **Agar-JSC (Petri dish)** | **SSF-JSC (flask)** | **Agar-CSC (Petri dish)** | **SSF-CSC (flask)** |
| INPA 1643 | *Stereum sp.* | 3.62 ± 0.49c | NG | 3.53 ± 0.57b | NG |
| INPA 1644 | *Schizophyllum commune* | 3.70 ± 0.12c | NG | 3.78 ± 0.68b | NG |
| INPA 1646 | *Coriolopsis sp.* | 7.37 ± 0.13a | 2.97 ± 0.34a | 6.96 ± 0.36[a] | 2.42 ± 0.28a |
| INPA 1668 | *Trametes sp.* | 3.66 ± 0.86c | NG | 3.47 ± 0.24b | NG |
| INPA 1681 | *Trametes sp.* | 3.63 ± 0.24c | NG | 3.70 ± 0.41b | NG |
| INPA 1689 | *Trametes sp.* | 3.66 ± 0.49 c | NG | 3.91 ± 0.67b | NG |
| INPA 1690 | *Trametes sp.* | 3.67 ± 0.93c | NG | 3.60 ± 0.11b | 1.98 ± 0.47a |
| INPA 1694 | NI | 3.61 ± 0.42c | 2.41 ± 0.65a | 3.71 ± 0.17b | NG |
| INPA 1695 | *Trametes sp.* | 3.55 ± 0.60c | 2.21 ± 0.71a | 3.91 ± 0.39b | 2.21 ± 0.39a |
| INPA 1696 | *Tyromyces sp.* | 3.83 ± 0.48c | 2.04 ± 0.43a | 3.85 ± 0.48b | 2.14 ± 0.34a |
| INPA 1698 | *Trametes elegans* | 1.92 ± 0.31e | 1.65 ± 0.74b | 2.38 ± 0.78c | 1.37 ± 0.17b |
| INPA 1719 | *Trametes sp.* | 3.36 ± 0.22c | 1.43 ± 0.32b | 3.70 ± 0.42b | NG |

| | | | | | |
|---|---|---|---|---|---|
| INPA 1720 | *Schizophyllum commune* | 3.68 ± 0.13c | NG | 3.65 ± 0.00b | 2.16 ± 0.41a |
| INPA 1725 | *Hexagonia hydnoides* | 3.73 ± 0.52c | 2.40 ± 0.48a | 3.72 ± 0.72b | 1.46 ± 0.23b |
| INPA 1734 | NI | 2.79 ± 0.26d | NG | 3.28 ± 0.50b | NG |
| INPA 1739 | *Flavodon flavus* | 7.04 ± 0.02b | 2.56 ± 0.48a | 7.31 ± 0.20a | 2.34 ± 0.49a |

NI: no-identified, NG: no-growth. Letters next to each metric value correspond to comparisons of the averages in the same column and not between one column and another (Tukey test $p \leq 0.05$). The fungi in bold were selected to continue the work.

**Tabela S2.** Literature results for degradation of phorbol esters in JSC, via solid-state fermentation by macrofungi.

| Fungus | Degradation rate | Fermentation time | Reference |
|---|---|---|---|
| *Pleurotus ostreatus* | 37% | 15 days | LUZ et al., 2013 |
| | 60% | 45 days | |
| | 99% | 60 days | |
| *Pleurotus ostreatus* | 58% | 15 days | KASUYA et al., 2012 |
| | 85% | 30 days | |
| | 99% | 45 days | |
| *Pleurotus sapidus* | 76% | 20 days | BOSE; KEHARIA, 2014 |
| *Pleurotus ostreatus* | 72.5% | | |
| *Pleurotus florida* | 67.9% | | |
| *Pleurotus sajor-caju* | 67.9% | | |
| *Ganoderma lucidum* | 100% | 20 days | BOSE; KEHARIA, 2014 |
| *Trametes zonata* | 100% | | |
| *Trametes gibbosa* | 91.7% | | |
| *Trametes versicolor* | 89% | | |
| *Trametes hirsuta* | 81.6% | | |
| *Phanerochaete chrysosporium* | 44.9% | | |
| *Phlebia rufa* | 97% | 30 days | BARROS *et al.*, 2011 |
| *Bjerkandera adusta* | 91% | | |
| *Ganoderma resinaceum* | 20% | | |

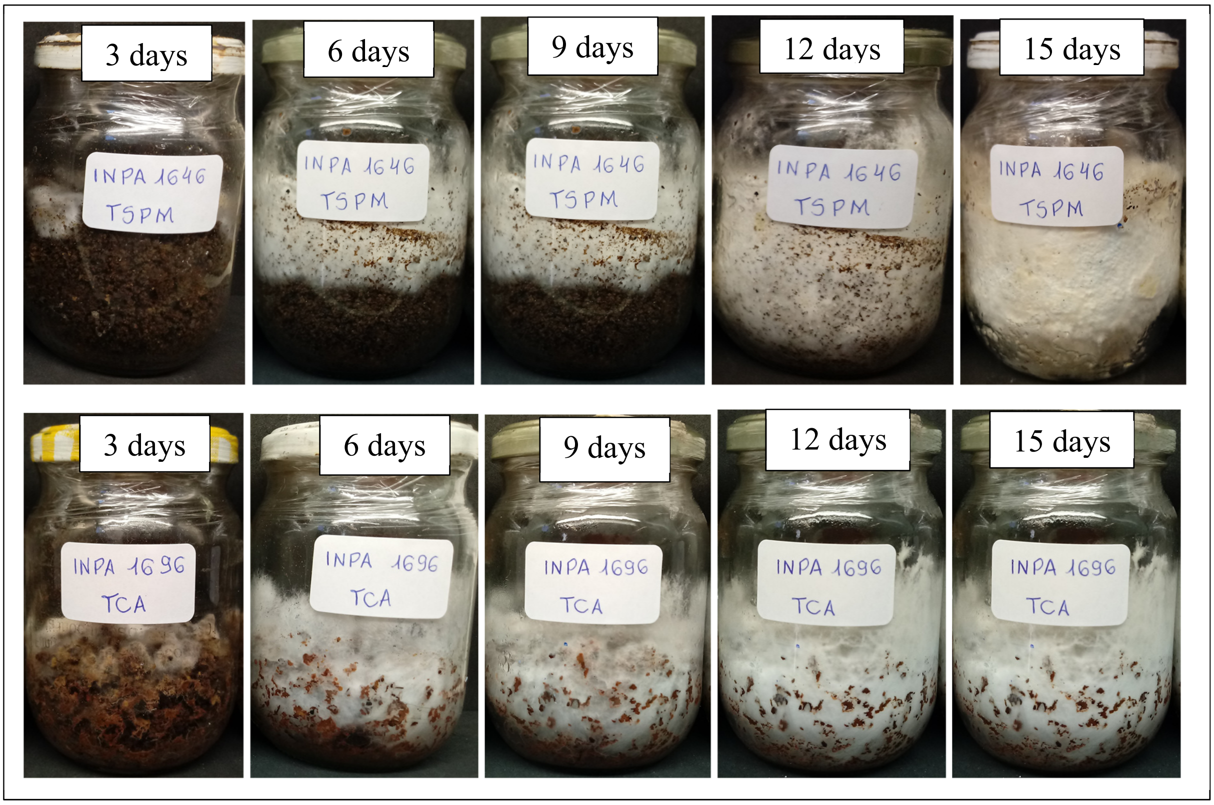


**Figure S1.** Pictures of the glass flasks containing the fermented cakes after 3, 6, 9, 12 and 15 days of incubation at 28°C, to illustrate the level of colonization of JSC and CSC by macrofungi *Coriolopsis sp.* and *Tyromices sp.*, respectively.

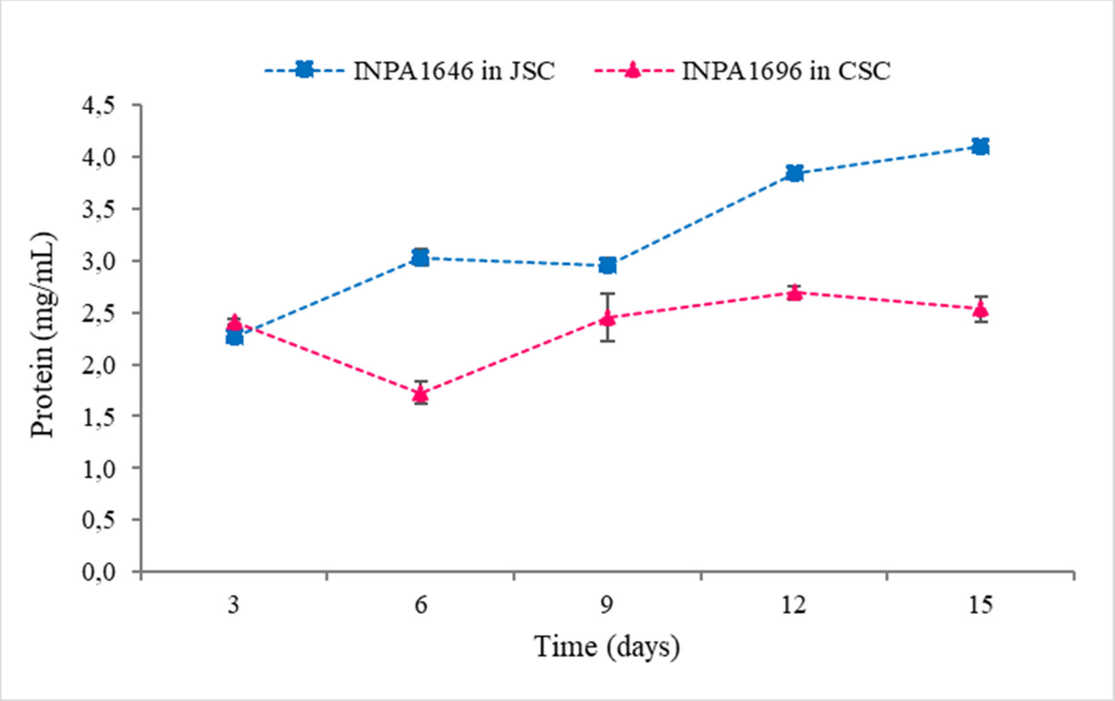


**Figure S2.** Total soluble protein kinetics in the crude extracts of SSF-JSC and SSF-CSC using *Coriolopsis sp*. and *Tyromices sp*., respectively.

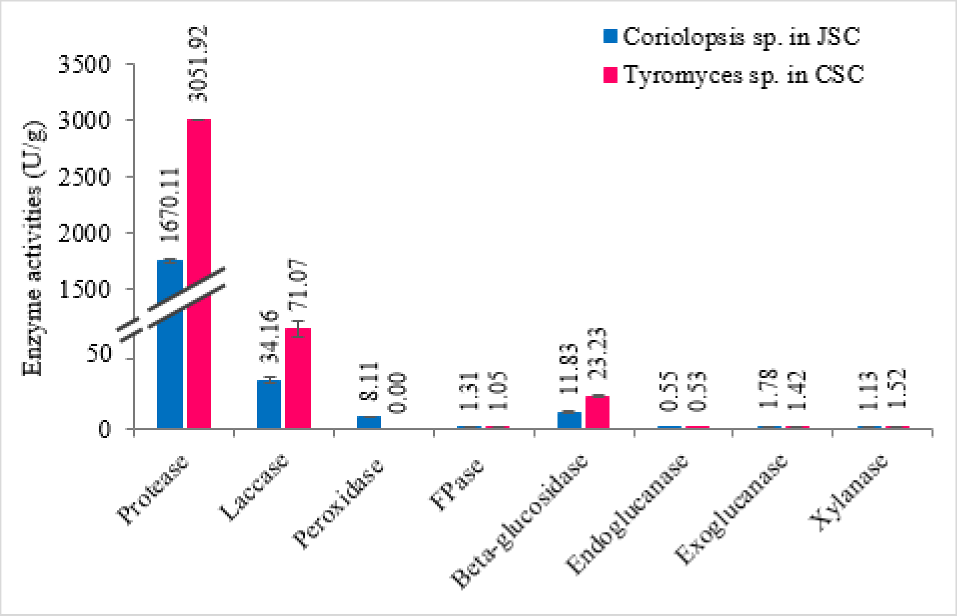


**Figure S3.** Total enzyme activities in the crude extracts of SSF-CSC and SSF-JSC using macrofungi *Tyromices sp*. and *Coriolopsis sp*., respectively, after 15 days of fermentation.
